# 'Exacerbation-free time' to assess the impact of exacerbations in patients with chronic obstructive pulmonary disease (COPD): a prospective observational study

**DOI:** 10.1038/s41533-018-0079-5

**Published:** 2018-04-03

**Authors:** L. M. Boer, E. W. Bischoff, X. Borgijink, J. H. Vercoulen, R. P. Akkermans, H. A. M. Kerstjens, W. J. Assendelft, T. R. Schermer

**Affiliations:** 1 Department of Primary and Community Care, Radboud Institute for Health Sciences, Radboud university medical center, Nijmegen, The Netherlands; 2 Department of Pulmonary Diseases, Groningen Research Institute for Asthma and COPD, University Medical Center Groningen, Groningen, The Netherlands; 3 Emergency Department, Gelre Hospitals, Zutphen, The Netherlands; 4 Department of Medical Psychology, Radboud Institute for Health Sciences, Radboud university medical center, Nijmegen, The Netherlands; 5 Department of Pulmonary Diseases, Radboud Institute for Health Sciences, Radboud university medical center, Nijmegen, The Netherlands; 6 Scientific Institute for Quality Department of Healthcare (IQ healthcare), Radboud Institute for Health Sciences, Radboud university medical center, Nijmegen, The Netherlands; 7 Faculty of Medical Sciences, University of Groningen, Groningen, The Netherlands; 8 Netherlands Institute For Health Services Research (NIVEL), Utrecht, The Netherlands

## Abstract

COPD exacerbations are commonly quantified as rate per year. However, the total amount of time a patient suffers from exacerbations may be stronger related to his or her disease burden than just counting exacerbation episodes. In this study, we examined the relationship between exacerbation frequency and exacerbation-free time, and their associations with baseline characteristics and health-related quality of life. A total of 166 COPD patients reported symptom changes during 12 months. Symptom-defined exacerbation episodes were correlated to the number of exacerbation-free weeks per year. Analysis of covariance was used to examine the effects of baseline characteristics on annual exacerbation frequency and exacerbation-free weeks, Spearman’s rank correlations to examine associations between the two methods to express exacerbations and the Chronic Respiratory Questionnaire (CRQ). The correlation between exacerbation frequency and exacerbation-free weeks was −0.71 (*p* < 0.001). However, among frequent exacerbators (i.e., ≥3 exacerbations/year, *n* = 113) the correlation was weak (*r* = −0.25; *p* < 0.01). Smokers had less exacerbation-free weeks than non-smokers (*β* = −5.709, *p* < 0.05). More exacerbation-free weeks were related to better CRQ Total (*r* = 0.22, *p* < 0.05), Mastery (*r* = 0.22, *p* < 0.05), and Fatigue (*r* = 0.23, *p* < 0.05) scores, whereas no significant associations were found between exacerbation frequency and CRQ scores. In COPD patients with frequent exacerbations, there is substantial variation in exacerbation-free time. Exacerbation-free time may better reflect the burden of exacerbations in patients with COPD than exacerbation frequency does.

## Chronic lung disease: The importance of exacerbation duration

Time spent exacerbation-free may provide a stronger indication of disease burden than exacerbation frequency for patients with chronic lung disease. Exacerbations in chronic obstructive pulmonary disease (COPD) are marked by a sudden decline in lung function, potential hospitalisation and a need to increase medication. Exacerbation frequency is used as a marker in COPD management, but this does not consider the duration of exacerbations or the impact this has on patients’ lives. Lonneke Boer at Radboud University Medical Center, the Netherlands, and co-workers questioned 166 patients every two weeks for a year about their experiences of exacerbation duration and frequency. There was substantial variation in exacerbation-free time between patients, with smokers most likely to suffer longer periods of poorer health. Exacerbation-free time was better correlated with health-related quality of life than exacerbation frequency.

## Introduction

Many patients with chronic obstructive pulmonary disease (COPD) experience exacerbations, which are defined as 'sustained worsenings of the patient’s condition from the stable state, and beyond normal day-to-day variation, that are acute in onset and necessitate a change in regular medication'.^1^ Exacerbations lead to accelerated decline in lung function,^2,3^ worse health status^3,4^ and increased health care utilisation,^5^ and are the main cause of COPD-related hospitalisations^6^ and mortality.^7^ In order to decrease the burden of exacerbations, prevention and prompt treatment of exacerbations are important goals in COPD patient management.^8^

Intervention studies in COPD often assess exacerbation frequency (i.e., a simple count of the number of exacerbation episodes within a certain time frame, usually a year) as a primary outcome measure. However, measuring just the frequency of exacerbations has limitations. First, the definitions and algorithms used to calculate exacerbation frequency vary considerably between studies, and the choice for a particular definition or algorithm may influence the results substantially.^9,10^ Also, exacerbation frequency may not be the most appropriate measure in every study. For example, interventions that focus on improving exacerbation self-management are not so much designed to prevent the occurrence of exacerbations, but to shorten the duration of an exacerbation and prevent its further escalation by early recognition and prompt treatment.^11,12^ Furthermore, exacerbation frequency is used in clinical settings to categorise COPD severity or to select patients for particular treatment options (for instance inhaled corticosteroids or long-acting bronchodilators), but is only moderately related to patient-reported outcomes such as quality of life.^13,14^

Duration of exacerbations is rarely used as an outcome measure, although it can vary widely between exacerbations and between patients.^2,15–17^ Donaldson and colleagues^2^ have shown that prolonged exacerbation recovery time leads to poorer health status and higher risk for subsequent exacerbations. Thus, to focus on duration of exacerbations instead of their frequency is an interesting concept, which may add to the understanding of the impact of exacerbations on patients with COPD. In a recent statement, the American Thoracic Society and European Respiratory Society encourage researchers to search for more patient-centred measures, since the relationship between physicians’ clinical goals and patients’ own priorities is often weak.^18^ From the patients’ perspective it may be more appropriate to look at the time they are free from exacerbations instead of looking at the time they are suffering from them.

In the current study, we explored how overall ‘exacerbation-free time’ relates to exacerbation frequency in patients with COPD. We also explored whether exacerbation-free time is related to patients’ baseline characteristics differently than exacerbation frequency is, and how these two methods to express exacerbations relate to patients’ health-related quality of life. We hypothesised that exacerbation-free time is only moderately correlated to exacerbation frequency, and that exacerbation-free time is more strongly related to health-related quality of life than exacerbation frequency is.

## Results

**Fig. 1:**
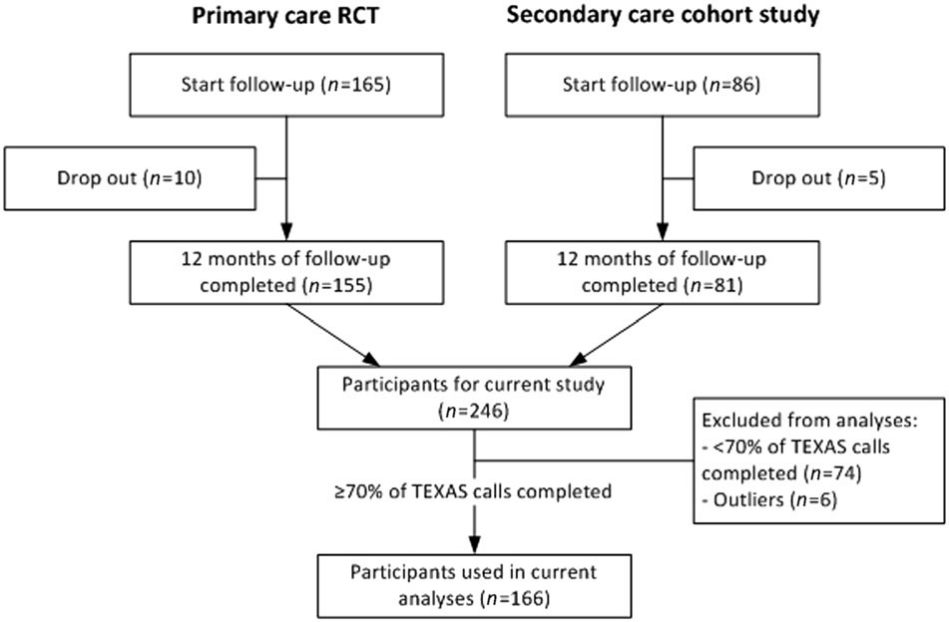
Flow chart of selection of patients for the current analysis from the two original studies. Abbreviation: TEXAS Telephonic EXacerbation Assessment System

Of the 246 participants in the two studies, 172 (69.9%) completed at least seventy percent of the TEXAS calls throughout their 12-month follow-up. Six patients were marked as outliers and excluded, leaving 166 patients for further analyses (see Fig. 1). Table 1 shows patients’ baseline characteristics for the two datasets combined and separately.

**Table 1:** Patient characteristics at baseline, presented in number (%) or mean (SD)

	Total sample (*n* = 166)	Primary care-RCT^20^ (*n* = 100)	Secondary care-cohort study^21^ (*n* = 66)
Patient characteristics			
Male (%)	119 (71.7)	67 (67.0)	52 (78.8)
Age (SD)	66.0 (8.6)	65.7 (8.8)	66.3 (8.4)
Current smokers (%)	45 (27.1)	35 (35.0)	10 (15.2)
BMI (SD)	26.3 (4.3)	26.6 (4.6)	25.9 (3.6)
FEV_1_% predicted (SD)	60.3 (18.0)	64.5 (16.7)	53.8 (18.2)
Obstruction severity (GOLD)			
Mild (%)	23 (13.9)	17 (17.0)	6 (9.1)
Moderate (%)	95 (57.2)	63 (63.0)	32 (48.5)
Severe (%)	41 (24.7)	18 (18.0)	23 (34.8)
Very severe (%)	7 (4.2)	2 (2.0)	5 (7.6)
MRC dyspnoea scale (*n* = 162)			
Category 1 (%)	63 (38.9)	37 (37.4)	26 (41.3)
Category 2 (%)	60 (37.0)	44 (44.4)	16 (25.4)
Category 3 (%)	39 (24.1)	18 (18.2)	21 (33.3)

*FEV*_*1*_ forced expiratory volume in 1 s, *GOLD* Global Initiative for Chronic Obstructive Lung Disease, *BMI* body mass index, *MRC* Medical Research Council

### Correlation between exacerbation frequency and exacerbation-free weeks

**Fig. 2:**
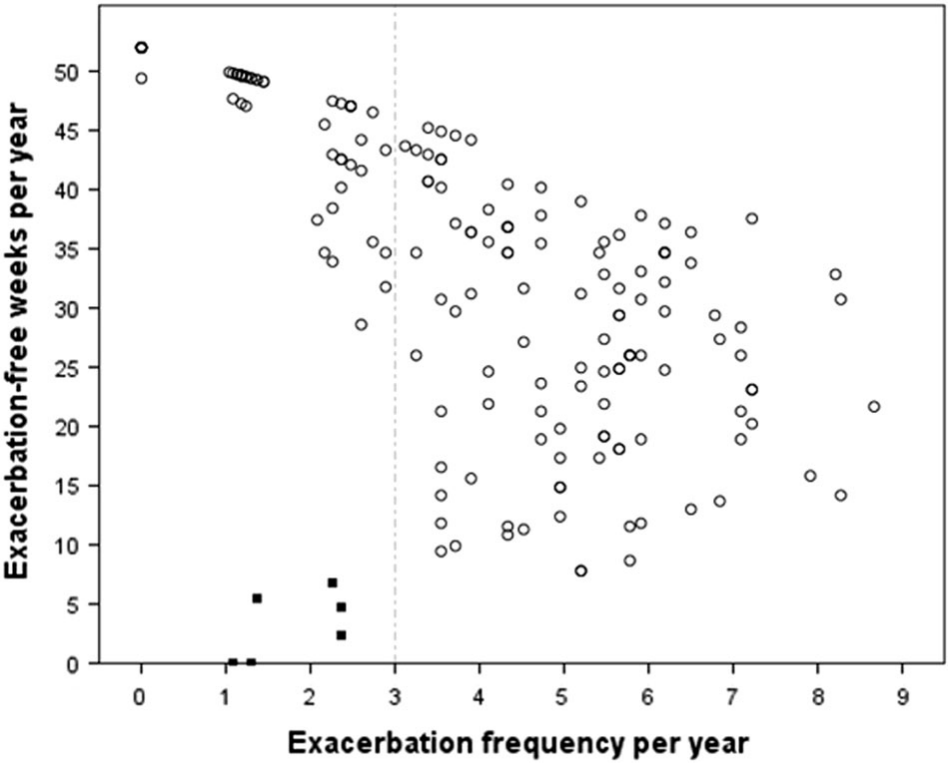
Scatter plot of exacerbation frequency (number of exacerbations/year) against exacerbation-free time (exacerbation-free weeks/year). Each dot represents one patient (*n* = 172). Solid dots: patients were considered as outliers and excluded from further analyses if they reported exacerbations for more than 85% of their follow-up time (*n* = 6). Dotted line: the study population was divided in infrequent (i.e., <3 exacerbations/year) and frequent (≥3 exacerbations/year) exacerbators, based on previous research^30^

Table 2 shows the mean number of exacerbations and exacerbation-free weeks per year. Pearson’s correlation between exacerbation frequency and exacerbation-free weeks was −0.71 (*p* < 0.001, 95% CI −0.77 to −0.62; Fig. 2). In the subgroup of infrequent exacerbators (i.e., those with <3 exacerbations/year, *n* = 53), the correlation between exacerbation frequency and exacerbation-free weeks was strong (*r* = −0.75, 95% CI −0.85 to −0.61; *p* < 0.001). Among the frequent exacerbators (≥3 exacerbation/year, *n* = 113) the correlation was weak (*r* = −0.25, 95% CI −0.42 to −0.07; *p* < 0.01).

**Table 2:** Self-reported exacerbation frequency and number of exacerbation-free weeks, presented in mean (SD) and number (%) of patients with frequent exacerbations (≥3/year)

	Total sample (*n* = 166)		Primary care-RCT^20^ (*n* = 100)		Secondary care-cohort study^21^ (*n* = 66)	
Number of TEXAS^a^ calls (SD)	21.3	(1.9)	21.0	(1.8)	21.6	(1.9)
Exacerbation frequency/year (SD)	4.0	(2.1)	4.0	(2.2)	4.1	(1.9)
Exacerbation-free weeks/year (SD)	33.4	(12.5)	33.8	(13.3)	32.8	(11.3)
Frequent (≥3) exacerbators (%)	113	(68.1)	68	(68.0)	45	(68.2)

*TEXAS* Telephonic EXacerbation Assessment System

^a^Calls were made every fortnight during the 12-month observation period

### Baseline characteristics in relation to exacerbation frequency and exacerbation-free weeks

Of the baseline characteristics analysed, only Medical Research Council (MRC) category was related to exacerbation frequency (main effect: *p* = 0.008). Table 3 shows the associations between MRC categories and exacerbation frequency and exacerbation free time. Patients in MRC category 1 (i.e., MRC score 0 or 1) had a significantly lower mean exacerbation frequency than patients in MRC category 3 (mean 3.4 ± 2.2 vs. 4.5 ± 2.0 exacerbations/year; *β* = −1.258, *p* = 0.006). No statistically significant difference between patients in MRC category 2 (mean 4.2 ± 1.8 exacerbations/year) and patients in MRC category 3 was observed (*β* = −0.320, *p* = 0.464). Exacerbation-free weeks were also related to baseline MRC (main effect: *p* = 0.043). Patients in MRC category 1 had significantly more exacerbation-free weeks than patients in MRC category 3 (mean 36.4 ± 12.9 vs. 28.6 ± 12.6 exacerbation-free weeks/year; *β* = 6.831, *p* = 0.014). No statistically significant difference between patients in MRC category 2 (mean 32.6 ± 11.7 exacerbation-free weeks/year) and patients in MRC category 3 was observed (*β* = 3.248, *p* = 0.228). Patients who were current smokers had less exacerbation-free time than patients who had quit smoking or had never smoked (mean 29.3 ± 13.3 vs. 34.7 ± 12.1 exacerbation-free weeks/year; *β* = −5.709, *p* = 0.014).

**Table 3:** Associations between baseline characteristics and exacerbation frequency and exacerbation-free weeks, analysed with two separate univariable analysis of covariance models (*n* = 166)

	Exacerbation frequency				Exacerbation-free weeks			
	*β*	95% CI		*p*	*β*	95% CI		*p*
Male	−0.230	−0.979	0.518	0.544	−2.151	−6.752	2.450	0.357
Age	−0.032	−0.072	0.009	0.121	0.026	−0.221	0.273	0.837
FEV_1_% predicted	−0.002	−0.021	0.017	0.866	0.060	−0.056	0.177	0.309
Current smoker	0.070	−0.670	0.809	0.853	−5.709	−10.252	−1.166	**0.014**
BMI	−0.032	−0.110	0.045	0.406	0.129	−0.345	0.602	0.591
MRC category 1^a^	−1.258	−2.144	−0.337	**0.006**	6.831	1.390	12.271	**0.014**
MRC category 2^a^	−0.320	−1.182	0.542	0.464	3.248	−2.049	8.544	0.228

*β* unstandardised Beta coefficients, *CI* confidence interval, *FEV*_*1*_ forced expiratory volume in 1 s, *BMI* body mass index, *MRC* Medical Research Council

^a^MRC category 3 (i.e., MRC score 3, 4 or 5) as reference category

Bold values are *p*-values below 0.05

### Exacerbation frequency and exacerbation-free time in relation to health-related quality of life

Table 4 shows that Spearman’s correlations between exacerbation frequency and CRQ scores at 12-month follow-up were weak. In contrast, correlations between exacerbation-free weeks and CRQ scores showed that having more exacerbation free time was related to a higher CRQ total score (*r* = 0.216, *p* = 0.034), better mastery (*r* = 0.217, *p* = 0.034), and less fatigue (*r* = 0.232, *p* = 0.023).

**Table 4:** Spearman’s rank correlation coefficients (*r*) of exacerbation frequency and exacerbation-free weeks with disease-specific HRQoL at 12-month follow-up (primary care cohort only, *n* = 96)

		Exacerbation frequency		Exacerbation-free weeks	
	Mean score (SD)	*r*	*p*	*r*	*p*
CRQ total	5.3 (0.80)	−0.052	0.614	**0.216**	**0.034**
CRQ emotions	5.4 (0.97)	−0.028	0.784	0.166	0.105
CRQ mastery	5.0 (0.66)	−0.035	0.738	**0.217**	**0.034**
CRQ fatigue	5.0 (1.17)	−0.069	0.506	**0.232**	**0.023**
CRQ dyspnoea	5.8 (1.15)	−0.079	0.446	0.185	0.071

*CRQ* Chronic Respiratory Questionnaire, *HRQoL* health-related quality of life

Bold values are *p*-values below 0.05

## Discussion

### Summary of the main findings

In this study we explored how COPD patients’ overall exacerbation-free time relates to the frequency of their exacerbations, analysed associations between baseline characteristics and these two ways of quantifying exacerbations, and assessed which of the two quantifications shows the strongest association with patients’ HRQoL. In research as well as in daily practice, looking at exacerbation-free time may be a valuable addition to exacerbation frequency when assessing the impact of exacerbations in patients with COPD, because we observed substantial variation in exacerbation-free time, particularly among patients who have frequent (i.e., three or more) exacerbations per year. We also found that patients who are current smokers have significantly less exacerbation-free time but not a higher annual exacerbation frequency than patients who do not smoke (29.3 vs. 34.7 exacerbation-free weeks, respectively). Our results showed no association between exacerbation frequency and COPD patients’ HRQoL, whereas more exacerbation-free time was related to better CRQ mastery, CRQ fatigue, and CRQ total scores.

### Comparison with existing literature

While several studies have examined variation in exacerbation length,^15,16^ time to next exacerbation,^15^ time to first hospitalisation,^19^ and the impact of interventions on exacerbation length,^12,20^ few studies in COPD have used exacerbation time per year (or the inverse, i.e. exacerbation-free time) as outcome measure. Donaldson et al.^2^ have examined the effect of exacerbation recovery time on health status. Our results seem consistent with their finding that longer exacerbation recovery time led to worse scores on the St. George Respiratory Questionnaire (0.20 units per one day longer recovery). Still, our findings differ on other aspects. For instance, our patients reported more time with exacerbations than the population of Donaldson’s London Cohort Study (35% vs. 9% of observation period, respectively).^2^ Also, our median exacerbation frequency is 4.1, whereas Donaldson et al. reported a median of 2.1. We have no clear explanation for these differences, since we had a heterogeneous study sample with a higher mean FEV_1_% predicted than the London cohort (60.3% vs. 45.7%, respectively) and we used the same definition of exacerbation.^2^ One difference between both studies is that the London Cohort Study made use of daily diaries where we made use of two-weekly telephone calls. Although this may account for some of the discrepancy in the time spent in exacerbations, our previous study demonstrated TEXAS to be a valid measure to assess exacerbation frequency.^21^ Our mean exacerbation frequency does correspond with the findings of Erdal et al.,^22^ who found a median exacerbation frequency of four in their hospital-recruited COPD patients.

Supporting smoking cessation is one of the key elements in the treatment of COPD,^8^ although the evidence for positive effects of smoking cessation on exacerbation frequency is weak.^23^ We found a statistically and clinically significant difference in exacerbation-free weeks between current smokers and ex-smokers that emphasises the importance of smoking cessation in patients with COPD.

We found no relationship between exacerbation frequency and HRQoL, which may seem in contrast to previous literature.^3,4^ However, these studies all used the SGRQ. This questionnaire measures several aspects of health status,^24^ but not specifically health-related quality of life.^25^ Our findings are in line with a previous study from our group, where we used the Nijmegen Clinical Screening Instrument to measure health status.^14^

### Strengths and limitations

A strength of this study was the large and heterogeneous study sample, derived from both a primary care and a hospital setting. Also, the collection of exacerbation-related data was very structured at the same date and time every week, with no possibility to skip questions, due to the use of our automated TEXAS system. However, although TEXAS has proven to be a valid system to capture exacerbations with two-weekly calls,^21^ for the measurement of exacerbation-free time, a timeframe of two weeks may not be ideal. Although the correlation between weekly and two-weekly data was very high (*r* = 0.90), we would have preferred more detailed measurement to assess exacerbation-free time, ideally using daily registrations. Nevertheless, the questions asked by TEXAS (see Appendix) explicitly refer to symptoms experienced during the last two weeks. Another limitation was that HRQoL was only assessed in the primary care cohort.

Inevitably, our approach and interpretation of missing data from calls will have influenced the number of exacerbations we found, as is the case in all studies.^9,10^ This limitation actually pleas in favour of using exacerbation-free time as an outcome measure; it seems more easy to establish when an exacerbation is not present than to establish when a new episode has exactly started. Furthermore, instead of introducing yet another instrument, we are proposing to use a new outcome that can be assessed with data that has already been collected in previous studies. Most studies using a symptom-based definition of exacerbations assess respiratory symptom changes with daily or weekly diaries. Exacerbation-free time can be calculated with these diaries also, so it would be an easy measure to add to analyses and published reports.

In conclusion, our findings indicate that exacerbation-free time and exacerbation frequency are two distinct quantifications to express exacerbations in patients with COPD, especially in frequent exacerbators. This, and the observed association between current smoking and exacerbation-free time is relevant for research as well as clinical practice. Although the correlation was weak, exacerbation-free time was associated with HRQoL, whereas exacerbation frequency was not. This supports our proposition that exacerbation-free time is a more patient-centred outcome than exacerbation frequency is. Further research is needed to examine if overall exacerbation-free time may be a sensitive measure to capture the more subtle effects of interventions aimed at improving self-management and prompt treatment of exacerbations. Health care providers should be aware that the time a patient suffers from exacerbations can vary widely among patients, may increase when the patient is an active smoker, and negatively influences COPD patients’ disease burden in terms of health-related quality of life.

## Methods

### Study design and population

Secondary analyses were performed on data from two studies that were previously conducted by our research group. The first study was a 12-month prospective cohort study in an outpatient setting in which exacerbation-related data were collected to validate the Telephonic EXacerbation Assessment System (TEXAS), an automated telephone system for assessing exacerbations in prospective studies.^21^ Recruitment took place in the Radboud university medical center in Nijmegen, the Netherlands in 2006 and 2007. Inclusion criteria relevant to the present analysis were: spirometry-confirmed COPD according to the GOLD criteria (i.e. ratio of post-bronchodilator forced expiratory volume in 1 s (FEV_1_) to forced vital capacity (FVC) <0.70); percentage of predicted FEV_1_ between 30 and 80%; and age ≥40. Patients with a severe comorbid condition with a reduced life expectancy were excluded.^21^

The second study was a 24-month, three-arm, multi-centre randomised controlled trial (RCT) in a primary care setting in which a COPD self-management programme was compared with programmed routine monitoring and usual care.^20^ Recruitment took place in 15 general practices in the eastern part of the Netherlands between 2004 and 2006. Inclusion criteria were post-bronchodilator FEV_1_/FVC <0.70 and age ≥35. Exclusion criteria were percentage of predicted FEV_1_ <30%; treatment by a chest physician; severe comorbid conditions with a reduced life expectancy; inability to communicate in the Dutch language (Clinical trials registration NCT00128765).^20^

For the present study, the two data sets were combined, leading to a mixed population of primary and secondary care patients with mild to severe COPD (Fig. 1).

Methods were performed in accordance with relevant regulations and guidelines. Both studies were approved by the Medical Ethics Committee (Arnhem-Nijmegen, the Netherlands; approval numbers 2006/081 and 2004/249, respectively). All patients gave written informed consent.

### Measurements and definitions

Sex, age, smoking history, body mass index, lung function, and the Medical Research Council (MRC) dyspnoea scale^26,27^ were assessed at baseline in both studies. The MRC dyspnoea scale is a single-item scale that measures functional limitations due to dyspnoea ranging from 0 (‘I never get breathless’) to 5 (‘I am too breathless to leave the house’).

Health-related quality of life (HRQoL) at baseline and 12 months was measured in the primary care RCT^20^ only, using the Chronic Respiratory Questionnaire (CRQ) self-administered standardised version.^28^ The CRQ is a 20-item questionnaire with four sub-domains: dyspnoea, fatigue, emotional function, and mastery. Items are scored on a seven-point scale, with higher scores indicating better HRQoL. A total score as well as separate domain scores are calculated.

#### Annual exacerbation frequency and exacerbation-free time

In both studies TEXAS^21^ was used to collect data on respiratory symptom aggravations. TEXAS called the patients every fortnight, asking standardised questions about changes in respiratory symptoms, respiratory medication use, and contacts with health care professionals in the previous two weeks (see Appendix 1). For every fortnight, an algorithm was used to calculate whether or not an exacerbation had occurred. Previously, in the prospective cohort study we established that exacerbation frequency could be measured validly using TEXAS every fortnight.^21^ To verify that the two-weekly TEXAS data could be converted to exacerbation-free weeks, the weekly conversion was correlated to the weekly paper diary cards that patients completed in that study to register exacerbation-related data. The correlation coefficient was very high (Pearson’s *r* = 0.90, 95% CI 0.86–0.94; *p* < 0.001).

In the absence of scientific consensus on a symptom-based definition, we adopted the most commonly used definition^9^ to determine whether or not exacerbations had occurred in a particular fortnight: a change for at least two consecutive days in two or more major symptoms (dyspnoea, sputum purulence, sputum amount), or a change in any one major symptom plus any one or more minor symptoms (common cold, wheeze, sore throat, and cough).^15,29^ After that, exacerbation-free time was defined as the inverse of exacerbation presence, i.e. the number of weeks without exacerbations (as defined above).

### Statistical analyses

To create two comparable data sets to merge into one, only the data from the first 12 months of the 24-month RCT^20^ were used. Patients were excluded from analysis if during the 12-month time frame <70% of TEXAS calls had been completed. For both exacerbation frequency and number of exacerbation-free weeks a mean number per year was calculated. Because TEXAS does not allow skipping questions, missing items within calls were not present. A call in which an exacerbation was scored (‘exacerbation positive’ call) was counted as a new episode when the previous call was ‘exacerbation negative’. When the previous call was ‘exacerbation positive’ the subsequent positive call was marked as an extension of the same exacerbation episode. When due to one or more missing calls it was unclear whether an ‘exacerbation positive’ call marked a new episode or was part of the previous exacerbation, it was marked as the same exacerbation episode.

A 99% confidence interval (CI) around the regression line was drawn in a scatter plot of exacerbation frequency (i.e., number of exacerbations/year) against exacerbation-free time (i.e., exacerbation-free weeks/year). Patients were considered as outliers and excluded from further analyses if they reported exacerbations for more than 85% of their follow-up time (see Fig. 2). To examine the relationship between exacerbation frequency and exacerbation-free weeks, two-sided Pearson’s correlation coefficients were calculated for the combined study population. Because the scatter plot showed an increasing variance in the number of exacerbation-free weeks when the annual exacerbation frequency increased, in a subgroup analysis, the study population was divided in infrequent (i.e., <3 exacerbations/year) and frequent (≥3 exacerbations/year) exacerbators. The cut-off was set at three exacerbations per year based on previous research.^30^ Two-sided Pearson’s correlations between exacerbation frequency and exacerbation-free weeks were calculated for each subgroup.

Next, associations between baseline characteristics (age, gender, smoking status, percentage of predicted FEV_1_, body mass index, and MRC dyspnoea score) and exacerbation frequency and exacerbation-free weeks were examined using two separate analyses of covariance models. Score 0, 4, and 5 of the MRC dyspnoea scale were infrequently reported (four, five, and three times, respectively). For statistical purposes, MRC scores 0 and 1 were recoded into category 1, score 2 into category 2 and scores 3, 4, and 5 into category 3. Finally, associations between exacerbation frequency and exacerbation-free weeks and the CRQ (total score and sub-domain scores) at 12 months of follow-up were examined using two-sided Spearman’s rank correlations.

*P* < 0.05 was considered statistically significant. IBM SPSS Statistics 22^©^ was used for all analyses.

### Data availability

The datasets generated during and/or analysed during the current study are available from the corresponding author on reasonable request.

## Electronic supplementary material

Appendix 1(PDF 424 kb)
