# Supplementary material for: 'Exacerbation-free time' to assess the impact of exacerbations in patients with chronic obstructive pulmonary disease (COPD): a prospective observational study
Source: NPJ Prim Care Respir Med. 2018 Apr 3;28:12. doi: 10.1038/s41533-018-0079-5 (PMC5882661; doi:10.1038/s41533-018-0079-5)
Supplement: Supplementary file 1 — Appendix 1(PDF 424 kb) [file 41533_2018_79_MOESM1_ESM.pdf]

Appendix 1

Content of TEXAS call (translated to English, original questions were in Dutch)*

Introduction:

The following questions concern your respiratory symptoms:

During the last two weeks, did you experience any events of **two or more days** on which ...

1.	… you were **shorter of breath** than usual?	**1**=YES / **2**=NO
2.	… you had **more sputum** than usual?	**1**=YES / **2**=NO
3.	… your **sputum** was **different in colour or composition** than usual?	**1**=YES / **2**=NO
4.	… you experienced **more wheezing** or your **chest felt tighter**?	**1**=YES / **2**=NO
5.	… you had a **sore throat**?	**1**=YES / **2**=NO
6.	… you had to **cough more** than usual?	**1**=YES / **2**=NO
7.	… you had a **cold or a runny nose**?	**1**=YES / **2**=NO
8.	… you used **more puffs** of your bronchodilator than usual?	**1**=YES / **2**=NO
9.	During the last two weeks, did you initiate a course of **prednisolone or antibiotics** because of a worsening of your respiratory symptoms?	**1**=YES / **2**=NO
10.	Did you experience days on which you were not able to perform your usual	**1**=YES / **2**=NO
10.b	On how many days did you experience this?	PRESS THE NUMBER OF DAYS AND END WITH **#**
11.	During the last two weeks did you need any unscheduled medical attention because of a worsening of your respiratory symptoms?	**1**=YES / **2**=NO
11.b	If so, did you visit your general practitioner (GP)?	press **1**
	Or did you visit an Emergency Department?	press **2**
	Or did you visit the GP after-hour service?	press **3**
	Or did you visit more than one of the above mentioned care providers?	press **4**
12.	Do you have any additional information or do you want to leave a	1= YES**/ **2**=NO

Thank you for answering this call. We will call you back in 14 days.

*** Patients received a laminated summary card with the precise questions and response categories for the TEXAS calls**

**** After pressing '1', patients were able to leave a short spoken message**
